# N-terminal phosphorylation of HP1α increases its nucleosome-binding specificity

**DOI:** 10.1093/nar/gku995

**Published:** 2014-10-20

**Authors:** Gohei Nishibuchi, Shinichi Machida, Akihisa Osakabe, Hiromu Murakoshi, Kyoko Hiragami-Hamada, Reiko Nakagawa, Wolfgang Fischle, Yoshifumi Nishimura, Hitoshi Kurumizaka, Hideaki Tagami, Jun-ichi Nakayama

**Affiliations:** 1 Graduate School of Natural Sciences, Nagoya City University, Nagoya 467-8501, Japan; 2 Laboratory of Structural Biology, Graduate School of Advanced Science and Engineering, Waseda University, Shinjuku-ku, Tokyo 162-8480, Japan; 3 Laboratory of Chromatin Biochemistry, Max Planck Institute for Biophysical Chemistry, 37077 Göttingen, Germany; 4 Proteomics Support Unit, RIKEN Center for Developmental Biology, Kobe 650-0047, Japan; 5 Graduate School of Medical Life Science, Yokohama City University, 1-7-29, Suehiro-cho, Tsurumi-ku, Yokohama 230-0045, Japan

## Abstract

Heterochromatin protein 1 (HP1) is an evolutionarily conserved chromosomal protein that binds to lysine 9-methylated histone H3 (H3K9me), a hallmark of heterochromatin. Although HP1 phosphorylation has been described in several organisms, the biological implications of this modification remain largely elusive. Here we show that HP1's phosphorylation has a critical effect on its nucleosome binding properties. By *in vitro* phosphorylation assays and conventional chromatography, we demonstrated that casein kinase II (CK2) is the kinase primarily responsible for phosphorylating the N-terminus of human HP1α. Pull-down assays using *in vitro*-reconstituted nucleosomes showed that unmodified HP1α bound H3K9-methylated and H3K9-unmethylated nucleosomes with comparable affinity, whereas CK2-phosphorylated HP1α showed a high specificity for H3K9me3-modified nucleosomes. Electrophoretic mobility shift assays showed that CK2-mediated phosphorylation diminished HP1α's intrinsic DNA binding, which contributed to its H3K9me-independent nucleosome binding. CK2-mediated phosphorylation had a similar effect on the nucleosome-binding specificity of fly HP1a and *S. pombe* Swi6. These results suggested that HP1 phosphorylation has an evolutionarily conserved role in HP1's recognition of H3K9me-marked nucleosomes.

## INTRODUCTION

Heterochromatin, a distinctive structure in the nucleus of eukaryotic cells, plays an important role in chromosomal function and epigenetic gene regulation. Heterochromatin is generally transcriptionally silent, and the assembly of higher-order chromatin structure is closely linked with changes in post-translational histone-tail modifications (1–3). In particular, the trimethylation of histone H3 lysine 9 (H3K9me3) is a hallmark of heterochromatin, and this modification is catalyzed by SUV39H-family histone methyltransferases. This mark serves as a binding site for a variety of chromatin proteins, including the evolutionarily conserved heterochromatin protein 1 (HP1) (4–6).

HP1 was originally identified in *Drosophila* as a factor enriched at condensed chromatin (7), and its homologs and isoforms have since been identified in diverse eukaryotic species, from fission yeast to humans (8–10). Mammalian cells express three HP1 isoforms, HP1α, HP1β and HP1γ. Although the cytological distribution of each isoform and the phenotypes of mice deficient for each of the isoforms suggest that they have distinct functions (11–15), their underlying molecular mechanisms have yet to be determined. While these HP1 isoforms are widely recognized to play primary roles in heterochromatin assembly, recent studies have revealed functions for them in cell-cycle control, transcriptional activation, DNA repair and other biological processes (16–19).

HP1-family proteins have two functionally distinct globular domains, the N-terminal chromodomain (CD) and the C-terminal chromoshadow domain (CSD), which are linked by an unstructured hinge region. The CD functions as a binding module that targets H3K9me3 marks (20–22). The CSD functions as a dimerization module that also provides an interaction surface for many other chromatin proteins involved in heterochromatin assembly and transcriptional regulation (23). The hinge region and the N- and C-terminally extended regions are unstructured and are less conserved than the CD or CSD, and are therefore thought to contribute to isoform-specific functions (8,24). HP1's ability to bind directly to chromatin and to form dimers suggests that it may bridge H3K9me3-marked nucleosomes to stabilize higher-order chromatin structure (25–27).

While the HP1 CD preferentially binds H3K9me3 peptides, the interaction is rather weak (21,22,28,29), and it is postulated that additional mechanisms contribute to HP1's nucleosome binding. Studies using reconstituted nucleosomes demonstrated that, although HP1 displays relatively lower binding specificity for H3K9me3-marked mononucleosome, the specificity increases when H3K9me3-marked oligonucleosomes are used as binding targets (27,30). Therefore, HP1's ability to bind H3K9me3-marked nucleosomes appears to be increased by its dimeric configuration, its higher-order intermolecular interactions or both (27). HP1's DNA- or RNA-binding activities may also increase its affinity for H3K9me3 nucleosomes. Studies in various organisms have shown that HP1 binds DNA and RNA through its hinge region, without any particular sequence specificity, and that several stretches of basic residues in the hinge region contribute to this binding (30–35). Since HP1's heterochromatic localization in mammalian cells is sensitive to RNase treatment (35,36), nuclear RNA may guide HP1 to its target heterochromatin. *In vitro* studies have demonstrated that DNA-binding activity associated with the hinge region contributes to HP1's preferential binding to nucleosomes with H3K9me3 (30) or with linker histones (32).

There is growing evidence that HP1-family proteins undergo a variety of post-translational modifications, including phosphorylation, acetylation, methylation, ubiquitination and SUMOylation (26,37). Phosphorylation, the most extensively studied of these modifications, is widely implicated in HP1's dynamics and functions. A metabolic labeling study revealed that mammalian HP1 proteins are subjected to phosphorylation in an isoform-specific manner (11). We previously showed that mouse HP1α is multiply phosphorylated at its N-terminal serine residues (S11–14), and that this phosphorylation enhances HP1α's affinity for the H3K9me3 peptide (29). In *Drosophila*, the casein kinase II (CK2) phosphorylation of an N-terminal serine (S15) of HP1a is involved in heterochromatic silencing (38,39). In *Schizosaccharomyces pombe*, the HP1 homolog Swi6 is phosphorylated by CK2, and a phosphorylation-defective Swi6 mutant leads to silencing defects and cannot recruit the histone deacetylase complex to chromatin (40). These findings suggest that the CK2-mediated phosphorylation of HP1 plays a critical role in heterochromatin assembly, although the mechanism by which phosphorylation controls HP1's functions remains unclear.

In this study, we found that HP1's phosphorylation plays a critical role in its nucleosome binding. Using *in vitro*-reconstituted nucleosomes, we demonstrated that N-terminal phosphorylation increased HP1α's binding specificity for H3K9me3-marked nucleosomes, and that this specificity was closely linked with HP1α's hinge-region-associated DNA-binding activity. Importantly, CK2-mediated phosphorylation had a similar effect on the nucleosome-binding specificities of fly HP1a and *S. pombe* Swi6. These results highlight an evolutionarily conserved role of HP1 phosphorylation in the assembly of higher-order chromatins.

## MATERIALS AND METHODS

### Cell culture

HeLa, HEK293T, U2OS and IMR90 cells were cultured in Dulbecco's modified Eagle's medium (Nacalai Tesque) supplemented with 10% fetal calf serum (Gibco). Plasmid DNA was transfected into HEK 293T cells using Lipofectamine 2000 Reagent (Invitrogen), and the cells were harvested after 24 h and used for experiments.

### Plasmid construction

Complementary DNAs (cDNAs) of human HP1α (NM_012117), HP1β (NM_006807) and HP1γ (BAA_83340) were polymerase chain reaction (PCR)-amplified from a HeLa cDNA library using SuperScript Reverse Transcriptase (Invitrogen) and a PfuTurbo PCR system (Agilent). The PCR products were cloned into the pCRII vector with the TOPO-TA Cloning Kit (Invitrogen), and then sequenced and subcloned into each expression plasmid. To produce recombinant proteins, the corresponding cDNAs were introduced into the pCold I vector (TaKaRa). Expression vectors for fly HP1a [NM_057407] (from S2 cells) and *S. pombe* Swi6 [NM_001018882] were constructed using the same method. To express full-length proteins in HEK293T cells, the corresponding cDNAs were introduced into pFLAG-C1 (29). The PfuTurbo PCR system was used for site-directed mutagenesis of the HP1α cDNA sequence. To express CK2 in *Escherichia coli* cells, mouse Ck2a and Ck2b (29) or *S. pombe* Cka1 and Ckb1 [NM_001019073 and NM_001020034] were cloned into pRSFDuet vectors (Novagen).

### Antibodies

The following antibodies were used in this study: anti-HP1α (MBL; BMP001), anti-HP1β (1MOD-1A9; Millipore), anti-HP1γ (2MOD-1G6; Millipore), anti-DmHP1a (sc-26950; Santa Cruz), anti-Swi6 (41), peroxidase-conjugated anti-histone H3 (ab21054; Abcam), anti-CK2α (1AD9; Calbiochem), anti-α-tubulin (T5168; Sigma), peroxidase-conjugated anti-FLAG M2 (A8592; Sigma), peroxidase-conjugated anti-rabbit immunoglobulin G (IgG; A6667; Sigma), peroxidase-conjugated anti-mouse IgG (112–035- 072; Jackson ImmunoResearch) and peroxidase-conjugated anti-goat IgG (A5420; Sigma).

### Immunoblotting with Phos-tag solution

Human cell lines were lysed in RIPA buffer (50 mM Tris-HCl [pH 8.0], 150 mM NaCl, 1% Nonidet P-40, 0.5% sodium deoxycholate and 0.1% sodium dodecyl sulphate (SDS)) supplemented with protease inhibitor cocktail (Complete [EDTA-free]; Roche) and 10 mM sodium fluoride (NaF). For dephosphorylation, 50 μl of whole-cell lysate (0.6 mg/ml) was diluted with an equal volume of 2× alkaline-phosphatase reaction buffer (100 mM Tris-HCl [pH 9.0], 10 mM MgCl_2_ and 60 units/ml shrimp alkaline phosphatase [SAP; TaKaRa]) and incubated for 3 h at 37°C. Polyacrylamide gel electrophoresis using a chemical reagent called Phos-tag (Wako) was performed as previously described (29).

### Production of recombinant HP1

Recombinant 6xHis-tagged proteins were expressed in BL21 (DE3) *E. coli* and purified by immobilized metal affinity chromatography following the manufacturer's instructions (TALON; Clontech). Recombinant HP1s were further purified by anion exchange chromatography (SOURCE 15Q; GE Healthcare). To produce CK2-phosphorylated HP1 proteins, BL21 (DE3) *E. coli* cells were simultaneously transformed with pCold I-HP1α (or -HP1a or -Swi6) and pRSFDuet-CK2α/β (-Cka1/Ckb1) plasmids and selected with ampicillin (for pCold I) and kanamycin (for pRSFDuet). The phosphorylated HP1 proteins were purified as described above. The experimental scheme for the recombinant phosphorylated protein preparation is shown in Supplementary Figure S4.

### Fractionation of cell extracts

HeLa cytoplasmic extracts (S100) and nuclear extracts (NE) were prepared following Dignam's protocol (42). These extracts were further separated on diethylaminoethyl (DEAE) Sepharose (GE Healthcare) for *in vitro* kinase assays. To identify the kinase responsible for phosphorylating the HP1α N-terminal domain, 10 mg of HeLa S100 or NE was loaded onto a HiTrap Q HP Column (GE Healthcare) equilibrated with buffer A (20 mM HEPES-KOH [pH 7.9], 10% glycerol, 0.2 mM EDTA, 0.5 mM phenylmethylsulfonyl fluoride and 0.5 mM dithiothreitol [DTT]). The bound proteins were eluted step-wise with a gradient from 0 to 1 M KCl in buffer A. After the *in vitro* kinase assay, fractions with kinase activity were pooled and further separated on a Superose 6 Column (GE Healthcare) equilibrated with buffer A containing 150 mM KCl.

### Kinase assays

For *in vitro* kinase reactions, 1 μg of recombinant HP1α-NCD (1–80 aa) was treated with 50 U of recombinant CK2 (NEB) or with 5 μl cell extract or column eluate in 25 μl kinase reaction buffer (20 mM Tris-HCl [pH 7.5], 50 mM KCl, 10 mM MgCl_2_ and 200 μM adenosine triphosphate) for 3 h at 30°C. To examine whether kinase activity was associated with CK2, 50 μM 4,5,6,7-tetrabromobenzotriazole (TBB) (Chemicon) was added to the reaction buffer.

### Immunopurification of FLAG-tagged HP1α from HEK293T cells

FLAG-tagged full-length HP1α proteins were transiently expressed in HEK293T cells. After 24 h, the cells were harvested, resuspended in immunoprecipitation (IP) buffer (50 mM HEPES-NaOH [pH 7.9], 0.3 M NaCl, 10% glycerol, 0.2 mM EDTA and 0.1% TritonX-100) and lysed by three freeze-thaw cycles. Cell lysates were clarified by centrifugation at 21 880 *g* for 30 min and subjected to immunoprecipitation using anti-FLAG M2 antibodies. Precipitated proteins were used for further experiments.

### Nucleosome reconstitution

Recombinant human histones H2A, H2B and H4 were overexpressed in *E. coli* cells as 6xHis-tagged proteins, and were purified as previously described (43). To install the H3K9me3 analog into the recombinant proteins, human histone H3.2 containing K9C and C110A mutations was expressed and purified as for the other histones. The purified H3_K9C/C110A protein was subjected to alkylation as previously described (44). A 193-bp nucleosome assembly 601 sequence DNA (601 DNA) and purified histones were mixed and reconstituted by salt dialysis as previously described (43). To obtain nucleosomes with biotinylated DNA, the same 193-bp 601 DNA was PCR-amplified using biotinylated primers. The following primers were used to amplify the 601 DNA: 193 bp_601_top (5′-ATC GGA CCC TAT CGC GAG CCA GGC CTG AGA-3′) and 193 bp_601_bot (5′-ATC TAT GAA TTT CGC GAC ACA AGG CCT GGA-3′).

### Nucleosome pull-down assays

Biotinylated nucleosomes (1 μg) containing either unmodified H3 or K9-trimethyl H3 (H3Kc9me3) were pre-incubated with streptavidin T1 Dynabeads (80 μg; Invitrogen) in binding buffer (10 mM Tris-HCl [pH 7.5], 150 mM NaCl, 0.1% Triton X-100, 5% glycerol, 0.05 mM EDTA, 0.1 mg/ml bovine serum albumin (BSA) and 1 mM DTT) for 4 h at 4°C. Beads were washed twice with the binding buffer to remove unbound nucleosomes. Nucleosome-bound beads were incubated with 100 pmol of recombinant HP1 proteins or with FLAG-HP1α purified from HEK293T cells for 1 h at 4°C in binding buffer. The beads were then triple-washed with washing buffer (10 mM Tris-HCl [pH 7.5], 300 mM NaCl, 0.1% Triton X-100, 5% glycerol and 0.05 mM EDTA), and the bound proteins were eluted by boiling the beads in SDS sample buffer. The input and bound proteins were resolved by SDS-polyacrylamide gel electrophoresis (SDS-PAGE) and analyzed by immunoblotting with cognate antibodies.

### 12-mer oligonucleosomal array preparation

12-mer oligonucleosomal arrays were prepared as previously described (45). In brief, 12× 601–200 DNA and assembled *Xenopus* histone octamers containing unmodified H3K9 or H3Kc9me3 were mixed in high-salt refolding buffer (10 mM Tris-HCl [pH 7.5], 2 M NaCl, 1 mM EDTA and 1 mM DTT), and the samples were dialyzed against 400 ml high-salt refolding buffer that was continuously replaced with 2 l low-salt (10 mM NaCl) refolding buffer over 24 h. The reconstituted samples were further dialyzed against 10 mM HEPES (pH 7.9), 0.1 mM EDTA and 1 mM DTT overnight.

### Chromatin coprecipitation assays

For each reaction, 1 μg unmodified or H3Kc9me3 12-mer oligonucleosomal arrays were diluted in 100 μl 1× binding buffer (10 mM Tris-HCl [pH 7.5], 150 mM NaCl, 5 mM MgCl_2_, 0.1 mM EDTA and 0.1% Triton X-100) and incubated with 1 μM HP1 proteins for 1 h on ice. The HP1-chromatin mixture was then centrifuged at 16 100 *g* for 30 min at 4°C. The pellet was washed with 500 μl 1× binding buffer and dissolved in SDS sample buffer. The 10% input and precipitated chromatin were analyzed by 15% SDS-PAGE and visualized by Coomassie brilliant blue (CBB) staining.

### Electrophoretic mobility shift assays (EMSAs)

Various concentrations of HP1 proteins were incubated with 0.2 pmol of 601 DNA in 10 μl of EMSA assay buffer containing 20 mM Tris-HCl (pH 7.5), 50 mM NaCl, 1 mM DTT and 0.1 mg/ml BSA for 15 min at 37°C. After incubation, 1 μl of 30% sucrose was added, and the samples were loaded onto 5% native polyacrylamide gels in 0.5× Tris-borate-EDTA. Gels were run at room temperature at 100 V for 1.5 h. Gels were stained with SYBR Gold (Invitrogen), visualized using a LAS3000 (GE Healthcare) and quantified using ImageQuant software. Binding curves were fitted with the following equation: fraction bound = 1 − fraction unbound. Curve-fitting was performed with Igor Pro software.

## RESULTS

### HP1α is constitutively phosphorylated at N-terminal serine residues in human cells

We previously showed that mouse HP1α is constitutively phosphorylated at its N-terminal serine residues (S11–14), and that this phosphorylation enhances HP1α's binding to H3K9me3 peptides (29). To address the conserved roles of HP1α phosphorylation, we examined the phosphorylation status of endogenous HP1α in several human cell lines using Phos-tag-PAGE, in which phosphorylated proteins migrate differentially (29).

**Figure 1.**
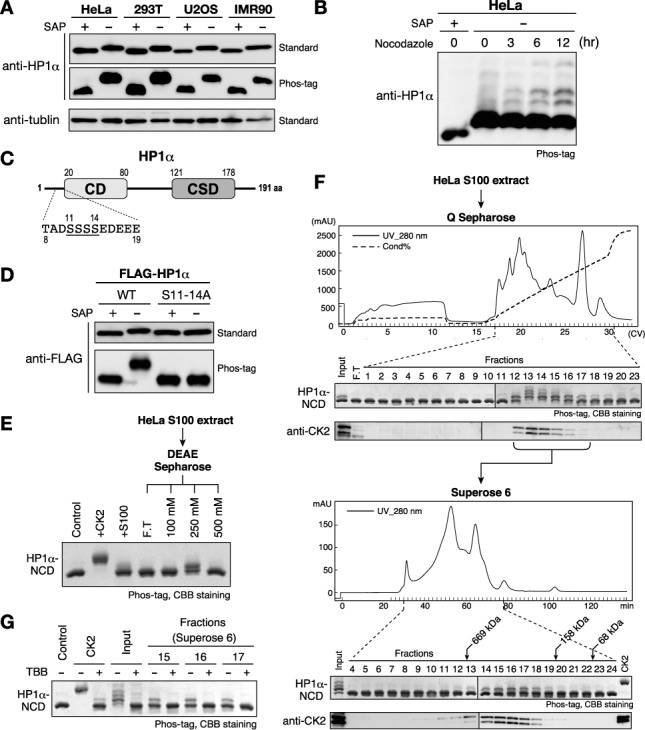
HP1α is constitutively phosphorylated by CK2 at N-terminal serine residues in human cells. (A) Phosphorylation states of HP1α in various human cell lines. SAP-treated samples were used as unphosphorylated basal control proteins. Samples were resolved on 12% standard SDS-PAGE gel (standard) or on 10% SDS-PAGE gel containing 50 μM Phos-tag (Phos-tag) gel, and analyzed by western blotting with an anti-HP1α antibody. Western blotting using an anti-α-tubulin antibody is shown for a loading control. (B) Phosphorylation patterns of human HP1α in G_2_/M-arrested cells. HeLa cells treated with 200 ng/ml nocodazole for 3, 6 or 12 h were harvested and analyzed as in (A). (C) Schematic diagram of the HP1α protein and its N-terminal amino acid sequence. Serine residues that are phosphorylatable by CK2 are underlined. (D) Serine clusters (S11–14) were constitutively phosphorylated *in vivo*. FLAG-tagged WT HP1α or HP1α with a S11–14A mutation was transiently expressed in HEK293T cells and analyzed as in (A). (E) *In vitro* kinase assays using recombinant protein containing the HP1α N-terminus and CD (HP1α-NCD). HeLa cytoplasmic extract (S100) and fractions that were partially purified through DEAE Sepharose were assayed. (F) Partial purification of cellular kinase(s) that phosphorylate HP1α-NCD: HeLa S100 extracts were loaded onto a HiTrap Q HP column, and each fraction was subjected to a kinase assay (top panels). Fractions containing kinase activity were pooled and further separated on a Superose 6 Column (bottom panels). Elution profiles of endogenous CK2 were analyzed by western blotting using an anti-CK2α antibody (beneath each panel). (G) Representative fractions in (E) were subjected to the *in vitro* kinase assay in the presence or absence of the CK2-specific inhibitor TBB.

Endogenous HP1α was detected as a single major band, which shifted slightly to a faster-migrating form after SAP treatment (Figure 1A). As previously observed for mouse HP1α, additional, slower-migrating minor bands appeared in nocodazole-treated (G2/M-arrested) HeLa cells (Figure 1B). The unphosphorylated form of HP1α was barely detected in any examined cells, suggesting that HP1α was constitutively phosphorylated in human cells. Amino acid substitutions in the N-terminal serine residues, S11–14A (Figure 1C) shifted the migration levels of the major band to that of the unphosphorylated control (Figure 1D), indicating that the major band shift of human HP1α was also attributable to its N-terminal phosphorylation (29).

### CK2 is the kinase primarily responsible for HP1α's N-terminal phosphorylation

As shown in Figure 1C, HP1α's N-terminal phosphorylation sites closely resemble the CK2's consensus target sequence (SXXE/D, SXE/D, SD) (46) and CK2 is known to phosphorylate the N-terminal serine residues of mouse HP1α *in vitro* (29). However, we could not obtain direct evidence for CK2's involvement in HP1α N-terminal phosphorylation: knocking down the CK2α/α’ catalytic subunit did not have any marked effect on murine HP1α's phosphorylation (Supplementary Figure S1A), and the potent CK2-specific inhibitor 4,5,6,7-TBB or tetrabromocinnamic acid did not clearly block the phosphorylation of endogenous murine HP1α (Supplementary Figure S1B) or transiently expressed FLAG-tagged human HP1α (Supplementary Figure S1C).

To identify the kinase or kinases responsible for HP1α's N-terminal phosphorylation, we conducted an *in vitro* assay in which a recombinant protein containing HP1α's N-terminus and CD (HP1α-NCD) was used as the substrate. Both HeLa cytoplasmic and nuclear extracts (S100 and NE) showed weak phosphorylation activity for HP1α-NCD, and this activity was enriched in the 250 mM KCl-eluted fractions from DEAE Sepharose (Figure 1E and Supplementary Figure S2A). We further monitored the enzyme activity through several chromatography columns (Figure 1F and Supplementary Figure S2B).

During the purification process, we found that the elution profile of the kinase activity was clearly overlapped with that of endogenous CK2 (Figure 1F and Supplementary Figure S2B). We further confirmed that the kinase activities in the partially purified preparations were abolished by adding TBB (Figure 1G). Although we could not exclude the possibility of minor contributions from other kinases, these results strongly suggested that the CK2 is primarily responsible for HP1α's N-terminal phosphorylation. Given that CK2 has a variety of substrates, it is likely that inhibiting CK2 led to defective cell proliferation before a clear reduction in HP1α N-terminal phosphorylation could be noted in the cells.

### HP1α's N-terminal phosphorylation increases its binding specificity for H3K9me3 nucleosomes

Previous studies have indicated that N-terminal phosphorylation enhances HP1α's binding to H3K9me3 peptides (29), but the effect of this phosphorylation on HP1α's binding to nucleosomes containing H3K9me3 was unclear. We addressed this issue using *in vitro*-reconstituted nucleosomes containing H3Kc9me3, prepared by installing a methyl lysine analog through the alkylation of a cysteine residue (Supplementary Figure S3), and examining the nucleosomes’ interaction with HP1 proteins in pull-down assays. The reconstituted nucleosomes were immobilized via biotinylated DNA on streptavidin-coated magnetic beads, and the bound HP1 proteins were evaluated by western blotting.

**Figure 2.**
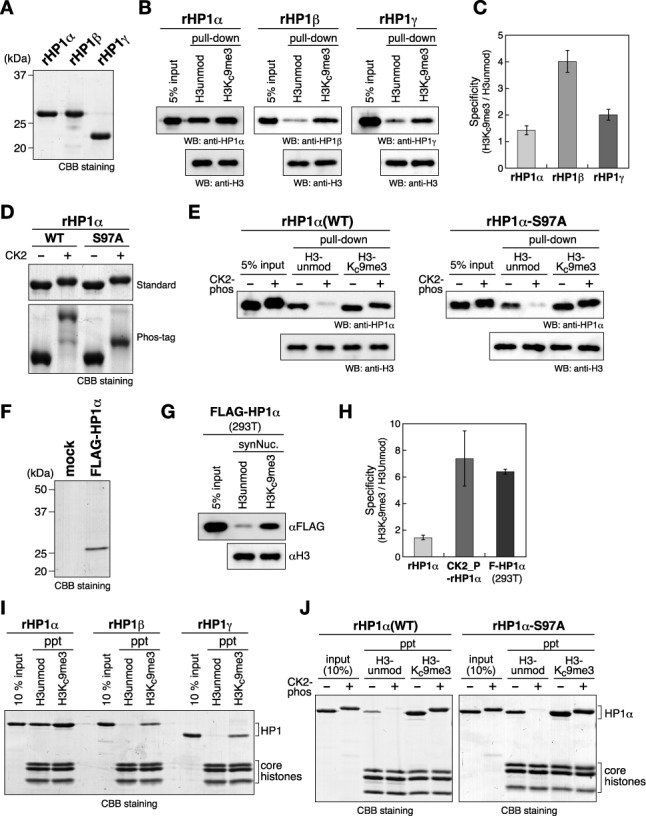
The N-terminal phosphorylation of HP1α increases its binding specificity for H3K9me3 nucleosomes. (A) Recombinant human HP1α, HP1β and HP1γ prepared from *E. coli*. (B) Representative nucleosome pull-down assays using synthesized nucleosomes containing unmodified H3 (H3unmod) or H3 with a K9me3 analog (H3Kc9me3), and biotinylated 193-bp 601 DNA. Recombinant human HP1α, HP1β or HP1γ was incubated with nucleosomes immobilized on streptavidin-beads, and the input and recovered proteins were resolved by SDS-PAGE and immunoblotted using cognate antibodies. (C) Quantified ratios of H3Kc9me3 nucleosome-bound HP1 over HP1 bound to H3unmod nucleosomes. Error bars represent the range of the standard deviations of three independent measurements. (D) Control and CK2-phosphorylated WT and S97A HP1α proteins were resolved by standard or Phos-tag PAGE. (E) Representative nucleosome pull-down assays using control and CK2-phosphorylated HP1αs. (F) FLAG-tagged HP1α (FLAG-HP1α) prepared from HEK293T. FLAG-HP1α transiently expressed in HEK293T cells was purified using anti-FLAG M2 agarose. A mock purification result is also shown (mock). (G) A representative nucleosome pull-down assay using HP1α prepared from HEK293T cells. (H) The quantified ratios of H3Kc9me3 nucleosome-bound HP1α over HP1α bound to H3unmod nucleosomes. Error bars represent the range of the standard deviations of three independent measurements. (I and J) Chromatin co-precipitation assays using 12-mer nucleosomal arrays. Recovered nucleosomes and bound HP1 proteins were analyzed by15% SDS-PAGE and visualized by CBB staining.

In the pull-down assay, recombinant human HP1α, HP1β and HP1γ (rHP1α, rHP1β and rHP1γ) produced in *E. coli* cells (Figure 2A) exhibited distinct binding preferences for the *in vitro*-reconstituted nucleosomes: rHP1β bound strongly to the H3Kc9me3 nucleosome and weakly to the control H3-unmodified (H3-unmod) nucleosome (Figure 2B), and although rHP1γ showed a similar binding preference, it appeared to bind H3-unmod nucleosomes more strongly than did rHP1β (Figure 2B). While rHP1α bound H3Kc9me3 nucleosomes, it also efficiently bound H3-unmod nucleosomes (Figure 2B). As shown in Figure 2C, rHP1α's binding specificity for the H3Kc9me-nucleosome was apparently lower than that of either rHP1β or rHP1γ.

We next prepared phosphorylated rHP1α using an *E. coli* coexpression system, in which two CK2 subunits were simultaneously expressed with rHP1α in *E. coli* cells to phosphorylate the rHP1α (29). The phosphorylated rHP1α was then purified by immobilized-metal-affinity- and anion-exchange chromatography (Supplementary Figure S4A). Western blotting analysis confirmed that there was no contaminating CK2 in the purified fractions (Supplementary Figure S4B). The phosphorylated HP1α was detected in a major and a minor band in Phos-tag PAGE (Figure 2D, WT). S97 in HP1α's hinge region fits well with the CK2 consensus sequence and, as previously demonstrated for mouse HP1α, the slow-migrating band disappeared when a single amino acid substitution, S97A, was introduced into rHP1α's hinge region (Figure 2D, S97A), suggesting that the faster-migrating band corresponded to N-terminally phosphorylated rHP1α, and that the slower-migrating rHP1α was phosphorylated at both S97 and at the N-terminal serine residues. Since HP1α species with S97 phosphorylation was barely detectable *in vivo* (Figure 1D), it may be blocked or efficiently removed by phosphatase(s) *in vivo*.

Phosphorylated wild-type (WT) rHP1α and control rHP1α bound H3Kc9me3-marked nucleosomes with comparable affinity (Figure 2E). Interestingly, however, once phosphorylated, the WT rHP1α's ability to bind H3-unmod nucleosomes was clearly weaker than that of the original rHP1α (Figure 2E). Phosphorylated rHP1α-S97A's binding of the H3-unmod nucleosome was also weak, suggesting that these changes in nucleosome binding were mainly due to the acquired N-terminal phosphorylation. Importantly, when phosphorylated, HP1α's binding specificity for H3Kc9me3-marked nucleosomes has become comparable to that of rHP1α prepared from 293T cells (Figure 2F–H).

Previous studies have shown that both human HP1α and the *S. pombe* HP1 protein, Swi6, display a lower specificity for the H3K9me3 mark in mononucleosomes than in H3-tail peptides, and that this specificity increases when assayed using nucleosome arrays (27,30). To further characterize the role of HP1α's N-terminal phosphorylation in its nucleosome binding, we examined HP1α's binding to a 12-mer nucleosome array with or without the H3Kc9me3 mark. In this experiment, reconstituted nucleosomes pre-incubated with HP1 in the presence of Mg^2+^ were precipitated by centrifugation, and the bound HP1 proteins were analyzed by SDS-PAGE.

As was the case for mononucleosomes (Figure 2B), rHP1β and rHP1γ bound efficiently to H3Kc9me3 oligonucleosomes and only negligibly to the H3-unmod control ones (Figure 2I). Although rHP1α's binding affinity was higher for H3Kc9me3 oligonucleosomes than for H3-unmod oligonucleosomes, it still robustly bound H3-unmod oligonucleosomes and this latter binding was clearly diminished by CK2-mediated phosphorylation (Figure 2J). A similar effect was observed for rHP1α-S97A, suggesting that N-terminal phosphorylation, but not S97 phosphorylation, plays a primary role in this differential binding. Taken together, these results suggested that the N-terminal phosphorylation of HP1α reduces its binding to H3-unmodified nucleosomes, and thereby increases its binding specificity for H3Kc9me3-marked nucleosomes.

### HP1α's N-terminal phosphorylation inhibits its ability to bind DNA

In contrast to our previous studies using histone peptides (29), our pull-down assays with *in vitro*-reconstituted nucleosomes showed that it has a distinct effect on HP1α's nucleosome binding. One of the differences between nucleosomes and free peptides is the presence of nucleosomal DNA. To investigate the relationship between HP1α's N-terminal phosphorylation and its DNA-binding activity, we conducted EMSAs using the free 193-bp 601 DNA that was used for reconstituting nucleosomes.

**Figure 3.**
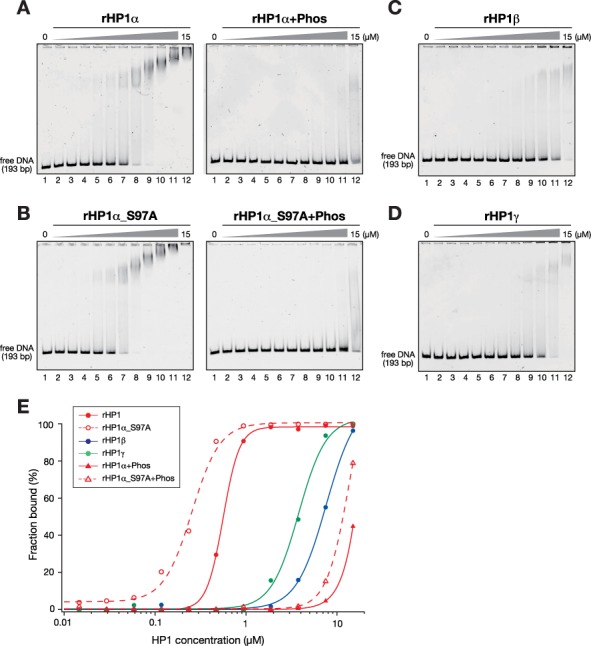
HP1α's N-terminal phosphorylation inhibits its DNA binding. (A and B) Representative EMSAs using control and CK2-phosphorylated, recombinant HP1α (rHP1α and rHP1α+Phos) (A) and HP1α with S97A mutations (rHP1α_S97A and rHP1α_S97A+Phos) (B). The HP1 concentration varied from 0 to 15 μM (0.5-fold dilutions). A 193-bp 601 DNA was used as a probe. Retarded probes in native PAGE gels were stained with SYBR Gold and detected by LAS3000. (C and D) Recombinant HP1β (C) and HP1γ (D) were subjected to EMSA as described in (A). (E) Quantification of EMSAs using HP1 proteins. The fraction of retarded DNA probe, determined by EMSA (A–D), was plotted against the HP1 protein concentration.

In this assay, rHP1α efficiently bound free DNA (Figure 3A). The retarded bands were broadly distributed, and migration levels changed gradually according to the dosage of rHP1α, implying that the binding ratio of DNA and rHP1α was not 1:1, but rather reflected that rHP1α multiply binds to a single DNA probe and/or that rHP1α mediates intermolecular DNA-DNA interactions. These assays showed that rHP1β and rHP1γ also bound DNA, but with much weaker affinity than rHP1α (Figure 3C–E). Interestingly, CK2-mediated phosphorylation clearly inhibited the DNA binding of rHP1α (Figure 3A and E). Since a similar inhibition was observed in rHP1α-S97A (Figure 3B and E), phosphorylation at the N-terminal, not at S97, appeared to play the primary role in this effect. These results were consistent with previous observations with fly HP1a (39), and further supported the concept that HP1α's intrinsic DNA-binding activity enhanced its H3K9me3-independent nucleosome binding, and the HP1α's N-terminal phosphorylation inhibited the DNA-binding activity.

### HP1α's N-terminal phosphorylation inhibits its DNA binding involving the hinge region

**Figure 4.**
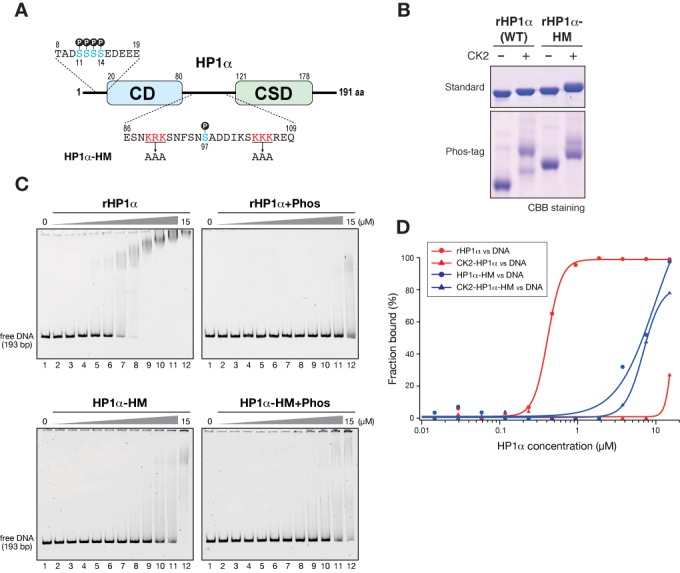
HP1α's N-terminal phosphorylation inhibits its hinge region-mediated DNA binding. (A) Schematic diagram of HP1α showing amino acid sequences of the N-terminal and hinge regions, and the positions of amino acid substitutions in an HP1α with a hinge mutation (HP1α-HM). Phosphorylatable serine residues are shown in blue, and the basic residues replaced by alanine in the HP1α-HM are shown in red. (B) Control and CK2-phosphorylated WT and hinge-mutated (HM) HP1α proteins were resolved by standard or Phos-tag PAGE and visualized by CBB staining. (C) Representative EMSAs using control and CK2-phosphorylated WT and HM HP1α. The HP1 concentration varied from 0 to 15 μM (0.5-fold dilutions). A 193-bp 601 DNA was used as a probe. (D) Quantification of EMSAs using HP1 proteins. The fraction of retarded DNA probe, determined by EMSA (C), was plotted against the HP1 protein concentration.

Previous studies have indicated that two stretches of basic residues in the hinge region are critical for HP1's ability to bind DNA (30,32). However, the N-terminal and hinge regions are physically separated by the CD, and the functional interactions between these regions have yet to be defined. To investigate whether N-terminal phosphorylation affects the HP1's DNA-binding mediated by the hinge region, we prepared mutant rHP1α carrying amino-acid substitutions in the hinge region (rHP1α-HM) (Figure 4A and B) and examined the effect of N-terminal phosphorylation on this mutant's DNA- and nucleosome-binding activities.

In SDS-PAGE gels, rHP1α-HM migrated slower than WT rHP1α, and the level of S97 phosphorylated species was lower than that of WT rHP1α (Figure 4B). It is likely that the substitution of charged amino acids affected rHP1α-HM's migration levels and CK2 activity for S97. Consistent with previous reports (30,32), amino acid substitutions at the hinge region greatly reduced HP1α's DNA-binding activity (Figure 4C, HP1α-HM). Importantly, however, phosphorylation only negligibly affected the DNA-binding activity of rHP1α-HM (Figure 4C, HP1α-HM+Phos). These results suggested that CK2-mediated phosphorylation inhibits DNA binding involving the hinge region. The DNA-binding activity of phosphorylated rHP1α-HM was slightly stronger than that of phosphorylated WT rHP1α (Figure 4D). Differences in the S97-phosphorylation state might affect residual DNA-binding activity, or the N-terminal phosphorylation might influence other parts of the molecule.

### Basic amino acid residues at the N-terminal tail modulate HP1's DNA-binding activity involving the hinge region

**Figure 5.**
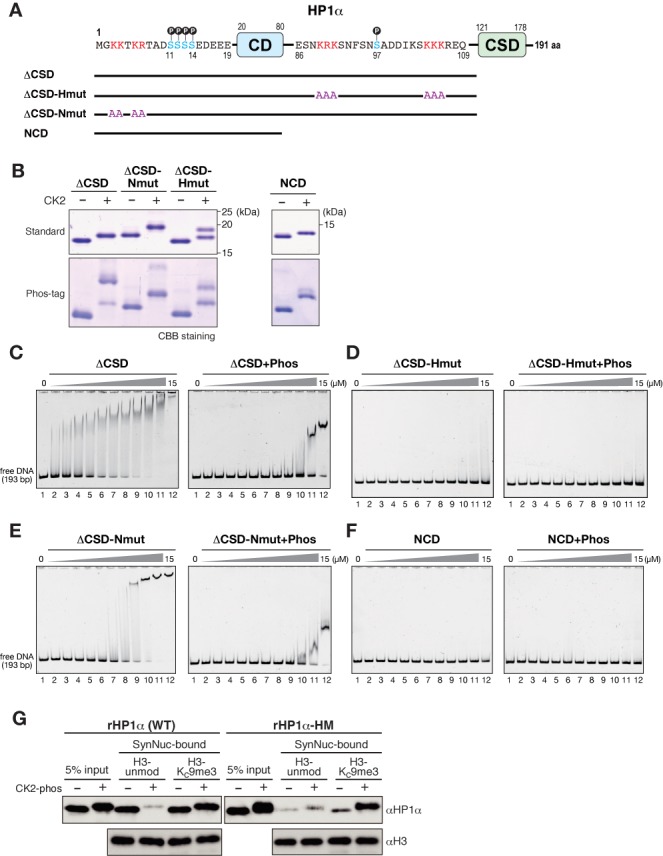
Basic amino acid residues at HP1α's N-terminal tail modulate DNA binding involving the hinge region. (A) Schematic diagram of full-length HP1α with the amino acid sequences of the N-terminal and hinge regions (top), and truncated ΔCSD mutants with amino acid substitutions. Phosphorylatable serine residues are shown in blue, and the basic residues replaced by alanine in hinge-mutated (Hmut) or N-terminal-mutated (Nmut) ΔCSD constructs are shown in red. (B) Control and CK2-phosphorylated, ΔCSD, ΔCSD-Nmut, CSD-Hmut and NCD proteins were resolved by standard or Phos-tag PAGE and visualized by CBB staining. (C–F) Representative EMSAs using control and CK2-phosphorylated ΔCSD (C), ΔCSD-Hmut (D), ΔCSD-Nmut and NCD mutants (F). The protein concentration varied from 0 to 15 μM (0.5-fold dilutions). A 193-bp 601 DNA was used as a probe. (G) Nucleosome pull-down assays using synthesized nucleosomes. Recombinant WT HP1α or HP1α with a HM were incubated with nucleosomes immobilized on streptavidin-beads, and the input and recovered proteins were resolved by SDS-PAGE and immunoblotted using an anti-HP1α antibody.

The results of amino acid substitutions at rHP1α's hinge region suggested that N-terminal phosphorylation suppresses the intrinsic DNA-binding activity mediated by the hinge region, and implied that these two domains interact both physically and functionally. Given that HP1 forms a stable dimer through the CSD, it is possible that the N-terminal and hinge domains interact intermolecularly or within monomeric rHP1α. To examine how N-terminal phosphorylation affects DNA binding involving the hinge region, we produced a CSD-truncated mutant HP1α (rHP1α-ΔCSD) and examined its DNA-binding activity and the effect of N-terminal phosphorylation in EMSAs (Figure 5A and B and Supplementary Figure S5A). Consistent with previous reports using HP1 proteins with a CSD mutation (41,47), rHP1α-ΔCSD behaved as a monomer in chromatographic analyses; no clear multimeric interaction was detected (data not shown).

Consistent with a previous observation (30), rHP1α-ΔCSD bound DNA more efficiently than WT HP1α (Figure 5C and Supplementary Figure S5B). This result suggested that HP1α was able to bind DNA without a dimeric configuration, and that the change in distribution pattern of retarded bands was mediated primarily by the binding of multiple rHP1α molecules to a single DNA probe. Interestingly, CK2-mediated phosphorylation greatly reduced rHP1α-ΔCSD's ability to bind DNA (Figure 5C and Supplementary Figure S5B). Since rHP1α-ΔCSD's DNA binding was also mediated primarily by the basic amino acids in the hinge region (Figure 5D and Supplementary Figure S5C), these results suggested that N-terminal phosphorylation inhibits DNA binding involving the hinge region of the same HP1α molecule.

Although the functional implications of HP1α's N-terminal region distal to S11–14 have yet to be determined, four basic amino acid residues are located in this region (Figure 5A). Basic amino acid residues are also present in the corresponding regions of HP1β and HP1γ (26), and a previous nuclear magnetic resonance (NMR) study suggested that these residues are involved in HP1β's binding to H3-unmod nucleosomes or DNA (24). Thus, we hypothesized that these N-terminal basic amino acid residues contribute to HP1α's binding of DNA. We tested this idea by introducing amino acid substitutions into the N-terminus of rHP1α-ΔCSD, and examining its DNA-binding activity in EMSAs (Figure 5A and B and Supplementary Figure S5A, Nmut).

The amino acid substitution of these N-terminal basic residues clearly reduced rHP1α-ΔCSD's ability to bind DNA, and changed the pattern of retarded DNA (Figure 5E and Supplementary Figure S5D). Importantly, CK2-mediated phosphorylation further reduced the DNA-binding ability of the rHP1α-ΔCSD mutant containing N-terminal substitutions. While N-terminal basic amino acid residues appeared to contribute to rHP1α's binding of DNA, almost no DNA binding activity was observed for rHP1α-ΔCSD with a hinge mutation (Figure 5D and Supplementary Figure S5C, ΔCSD-Hmut) or for rHP1α-NCD (Figure 5F). Together, these results suggested that although HP1α's N-terminal region could not stably bind DNA by itself, the hinge region's DNA-binding activity was enhanced by basic residues in the N-terminus, and was strongly reduced by phosphorylation at the proximal N-terminal serine residues.

### Dual role of HP1α N-terminal phosphorylation in binding H3K9me3 nucleosomes

Our results presented thus far indicate that the role of N-terminal phosphorylation is to suppress the hinge region's intrinsic DNA-binding activity. To clarify the functional importance of N-terminal phosphorylation in HP1α's nucleosome binding, we performed a nucleosome pull-down assay using rHP1α-HM, which retained only a weak DNA-binding activity (Figure 4C and D).

The nucleosome pull-down assay showed that rHP1α-HM bound H3-unmod nucleosomes only negligibly, regardless of its phosphorylation state (Figure 5G), supporting the idea that rHP1α's association with H3-unmod nucleosomes was mediated by its DNA-binding activity. Interestingly, the hinge mutation also greatly reduced rHP1α's binding of H3Kc9me3 nucleosomes (Figure 5G). In contrast, phosphorylated rHP1α-HM robustly bound H3K9me3-marked nucleosomes. Although we could not rule out the possibility that the hinge mutation somehow affects CD's interaction with H3Kc9me3 tail, these results suggested that unphosphorylated rHP1α's binding of H3Kc9me3 nucleosomes (Figures 2B, E, and 5G) was mediated, at least in part, by its DNA-binding via the hinge region. These results also suggested that, without CK2-mediated phosphorylation, the CD's H3Kc9me3-binding activity was not sufficient to establish a stable interaction between rHP1α and H3Kc9me3-nucleosomes (Figure 5G). As we showed previously (29), it was likely that CK2-mediated phosphorylation enhanced CD's H3Kc9me3-binding activity, which contributed to the rHP1α-HM's H3Kc9me3 binding. Together, these results strongly suggested that the N-terminal phosphorylation of HP1α plays dual roles, simultaneously promoting the H3K9me3 binding and suppressing the intrinsic DNA-binding activity.

### Conserved role of CK2-mediated phosphorylation in HP1's nucleosome binding

**Figure 6.**
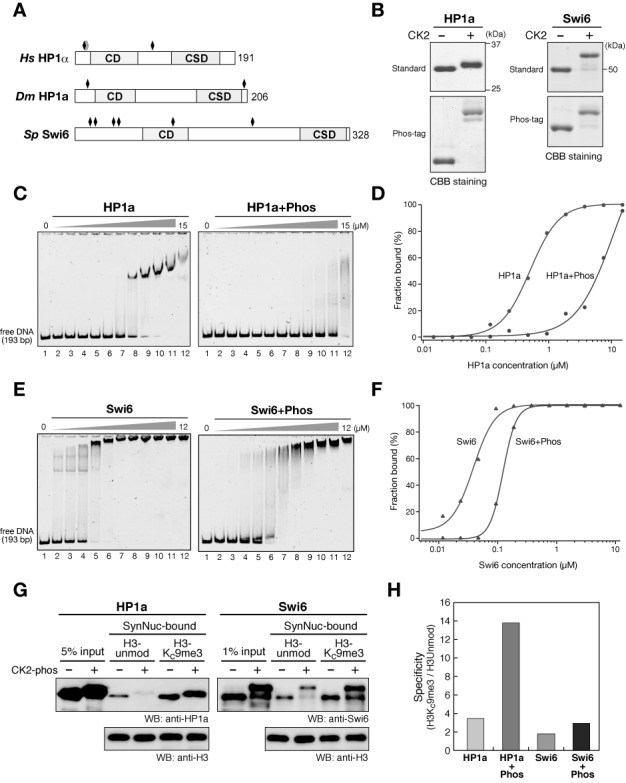
Conserved role of CK2-mediated phosphorylation in HP1's nucleosome binding. (A) Schematic drawings of human (*Hs*) HP1α, *Drosophila* (*Dm*) HP1a and *S. pombe* (*Sp*) Swi6. The positions of CK2-phosphorylatable serine residues are indicated by black rhomboids. (B) Control and CK2-phosphorylated HP1a and Swi6 were resolved by standard or Phos-tag PAGE and visualized by CBB staining. (C and E) Representative EMSAs using control and CK2-phosphorylated HP1a (C) and Swi6 (E). The protein concentration varied from 0 to 15 μM for HP1a, and from 0 to 12 μM for Swi6, respectively (0.5-fold dilutions). A 193-bp 601 DNA was used as a probe. (D and F) Quantification of EMSAs using HP1 proteins. The fraction of retarded DNA probe in (C) and (E) was plotted against the HP1 protein concentration. (G) Nucleosome pull-down assays using synthesized nucleosomes. Control and CK2-phosphorylated HP1a (left) and Swi6 (right) were incubated with nucleosomes immobilized on streptavidin beads, and the input and recovered proteins were resolved by SDS-PAGE and immunoblotted using cognate antibodies. (H) The quantified ratios of H3Kc9me3 nucleosome-bound HP1a or Swi6 over that bound to unmodified nucleosomes.

Although CK2 phosphorylates HP1 proteins in flies and fission yeast (Figure 6A) (38,40), the physiological role of CK2-mediated phosphorylation in these systems is unclear. CK2-phosphorylated sites have been mapped in these HP1 proteins’ N-terminal regions (Figure 6A), and the N-terminal phosphorylation of fly HP1a was shown to affect its ability to bind DNA (39). Therefore, CK2-mediated phosphorylation might play similar roles in HP1's nucleosome-binding activities in other organisms. To test this possibility, we used the CK2-coexpression system to phosphorylate fly HP1a and *S. pombe* Swi6, in which fly HP1a was phosphorylated by human CK2, whereas *S. pombe* CK2 was used for Swi6 (Figure 6B), and analyzed these proteins in EMSAs and nucleosome pull-down assays.

In accordance with previous results (39), fly HP1a efficiently bound DNA, and CK2-mediated phosphorylation strongly inhibited this binding (Figure 6C and D). Swi6's DNA-binding activity was much stronger than that of human HP1α or fly HP1a (Figure 6E), presumably due to its length and large number of basic residues. Although CK2-mediated phosphorylation reduced Swi6's ability to bind DNA, its DNA-binding activity was still relatively strong (Figure 6E and F). In the nucleosome pull-down assay, unphosphorylated fly HP1a preferentially bound H3K9me3 nucleosomes but clearly bound H3-unmod nucleosomes as well. Importantly, the CK2-mediated phosphorylation of fly HP1a diminished its binding of H3-unmod nucleosomes, which increased its specificity for H3Kc9me3 nucleosomes (Figure 6G).

As with fly HP1a, Swi6 bound both H3-unmod and H3Kc9me3 nucleosomes but preferred the latter target. Although CK2-mediated phosphorylation slightly increased Swi6's binding specificity for H3Kc9me3 nucleosomes, the effect was very small compared to that in human HP1α or fly HP1a (Figures 2H and 6H), and phosphorylated Swi6 could still stably bind H3-unmod nucleosomes (Figure 6G). This was presumably because Swi6 retained relatively strong DNA-binding activity even when phosphorylated (Figure 6E and F). Since Swi6 is heavily phosphorylated *in vivo* (40), it is possible that our coexpression system could not perfectly mimic Swi6's phosphorylation state *in viv*o, or that kinases other than CK2 provide additional phosphorylation that enhances Swi6's binding specificity for nucleosomes. While further studies are needed to clarify the role of Swi6's phosphorylation in its nucleosome binding, these results demonstrated that CK2-mediated phosphorylation suppresses the intrinsic DNA-binding activity of HP1 proteins in diverse organisms, and modulates their nucleosome binding.

## DISCUSSION

Although the DNA-binding activity of HP1-family proteins has been recognized for a long time, its implication in HP1's function has remained obscure. The present study demonstrated that human HP1α's N-terminal phosphorylation reduces its intrinsic DNA binding, which enhances its binding specificity for H3K9me3 nucleosomes. Our results further showed that CK2-mediated phosphorylation potentially affects HP1's function in other species. This study provides a novel insight into mutual interactions among HP1's phosphorylation, DNA-binding activity and nucleosome-binding specificity, all of which contribute to its function.

Previous studies have described the intrinsic DNA- or RNA-binding activities of HP1 proteins in various species. It should be noted that in mammals, such DNA-binding activity has been previously described only for HP1α, but not for HP1β or HP1γ (25,30,33,35). This is presumably due to HP1α's stronger DNA-binding activity compared with the other isoforms (Figure 3). Similarly, isoform-specific DNA binding has been described for *Xenopus* HP1 proteins: HP1α, but not HP1γ, strongly binds DNA (32). In this report, we demonstrated that human HP1α is constitutively phosphorylated by CK2 and, importantly, that this phosphorylation strongly inhibits HP1α's ability to bind DNA. Considering these results, the functional implications of HP1α's DNA-binding activity should be reassessed, as well as that of other HP1 proteins. *Xenopus* HP1α, which has strong DNA-binding activity, also contains serine clusters at its N-terminus, which fits perfectly with the CK2 consensus sequence. It is therefore likely that many of *Xenopus* HP1α's cellular functions are also regulated by its CK2-mediated phosphorylation. In addition, transient or constitutive phosphorylations may also affect the ability of mammalian HP1β or HP1γ to bind DNA.

While earlier studies using bulk nucleosomes demonstrated that HP1 could interact with nucleosomes independently of an H3 tail (12,34), our results suggest that HP1α's H3K9me3-independent nucleosome binding is primarily mediated by its DNA binding, which is consistent with another recent study (30). Importantly, this present study further demonstrates that HP1α's H3K9me3-independent nucleosome binding is dramatically reduced by CK2-mediated phosphorylation (Figure 2). In addition, we previously showed that the N-terminal phosphorylation of HP1α also contributes to the CD's interaction with the H3K9me3 tail (29). Therefore, it is likely that HP1α's nucleosome binding *in vivo* is more specific than deduced from earlier *in vitro* studies.

Consistent with recent studies using reconstituted H3K9me3-marked oligonucleosomes (27,30), we also found that HP1α's binding specificity was increased by using 12-mer nucleosomes (Figure 2I). One simple interpretation of this result would be that the presence of multiple H3K9me3 marks induced efficient HP1α's binding. Another possibility would be that reconstituted oligonucleosomes formed a compacted structure, which might restrict HP1α's DNA binding. Although it is unclear how HP1 binds to nucleosomal DNA, linker DNAs may be the preferential binding site of HP1. It is possible that compacted oligonucleosomes structure changed the flexibility of linker DNA and thereby inhibited HP1α's H3K9me3-independent binding.

HP1α's DNA-binding activity negatively affects its H3K9me3-specific nucleosome binding, and thus appears to contribute little to HP1α's function in nucleosome assembly. However, N-terminal-phosphorylated HP1α retained residual DNA-binding activity, and both HP1β and HP1γ possess such activity, albeit with weaker affinities. Considering that the patches of basic amino acid residues in the hinge region are highly conserved among HP1-family proteins, it is possible that DNA-binding contributes to the HP1 protein's initial interaction with the nucleosome, regardless of its H3 methylation state, and that HP1's CD subsequently recognizes H3K9me3 marks on the nucleosome. Alternatively, HP1's DNA-binding activity may contribute to isoform-specific functions. In contrast to the clear heterochromatic localization of HP1α and HP1β, HP1γ tends to localize to euchromatic regions as well to heterochromatin (11,12). Since HP1γ's ability to bind DNA is stronger than that of HP1β or CK2-phosphorylated HP1α (Figure 3), HP1γ's DNA-binding activity may contribute to its euchromatic localization. Another intriguing possibility is that the physiological targets of HP1 proteins are cellular RNAs rather than nucleosomal DNA (35,36). Although the effect of HP1α's phosphorylation on its RNA-binding activity has not been examined, it is plausible that HP1 tethers cellular RNAs after making stable contact with H3K9me3 nucleosomes.

In this study, we demonstrated that HP1α binds to DNA through at least two distinct regions, the hinge and the N-terminal regions. The CK2-mediated N-terminal phosphorylation of HP1α appears to interfere directly with the function of the N-terminal tail. Although an NMR study demonstrated that basic residues at HP1β's N-terminal tail contribute to its binding of DNA or unmodified nucleosomes (24), it is not clear how these regions interact structurally with each other. Since no multimeric interaction was detected for HP1α-ΔCSD, our EMSA analyses using HP1α-ΔCSD proteins (Figure 5) suggested that these domains work cooperatively within the same molecule. It is possible that the DNA-binding activity associated with these regions loosely fixes the HP1 protein's position on the nucleosome. Further structural studies are needed to define the interactions between HP1 and nucleosomes.

Regarding the physiological role of HP1α's CK2-mediated phosphorylation, we previously demonstrated that a mutant HP1α with deficient N-terminal phosphorylation showed impaired heterochromatic localization and caused increased genome instability. Although at the time we considered this effect to be due mainly to the HP1α mutant's inability to bind H3K9me3 (29), the results of the present study suggest that strong DNA- or RNA-binding ability could also interfere with the HP1α mutant's heterochromatin targeting. Previous studies using fly HP1a and fission yeast Swi6 demonstrated defective heterochromatic silencing in cells expressing a phosphorylation-deficient mutant HP1 (39,40). These phenotypes might also be explained by the mutant's increased DNA-binding activity or decreased H3K9me3-binding or both. Since unphosphorylated HP1α species were not detected in the mouse and human cells examined thus far (Figure 1), CK2-mediated phosphorylation may provide structural properties that cannot be replaced by other acidic amino acids, and may thus be closely linked with HP1 functions in diverse species. Such functional contributions of phosphorylation, whether mediated by CK2 or other cellular kinases, may also affect other chromatin-binding proteins.

## SUPPLEMENTARY DATA

Supplementary Data are available at NAR Online.

SUPPLEMENTARY DATA
