# Supplementary material for: N-terminal phosphorylation of HP1α increases its nucleosome-binding specificity
Source: Nucleic Acids Res. 2014 Oct 20;42(20):12498–511. doi: 10.1093/nar/gku995 (PMC4227797; doi:10.1093/nar/gku995)
Supplement: SUPPLEMENTARY DATA [file supp_42_20_12498__index.html]

N-terminal phosphorylation of HP1α increases its nucleosome-binding specificity — SUPPLEMENTARY DATA 

# N-terminal phosphorylation of HP1α increases its nucleosome-binding specificity

## SUPPLEMENTARY DATA

**Files in this Data Supplement:**

- SUPPLEMENTARY DATA
