# Supplementary material for: N-terminal phosphorylation of HP1α increases its nucleosome-binding specificity
Source: Nucleic Acids Res. 2014 Oct 20;42(20):12498–511. doi: 10.1093/nar/gku995 (PMC4227797; doi:10.1093/nar/gku995)
Supplement: SUPPLEMENTARY DATA [file supp_gku995_nar-02008-m-2014-File008.doc]

**N-terminal phosphorylation of HP1α increases its nucleosome-binding specificity**

Gohei Nishibuchi, Shinichi Machida, Akihisa Osakabe, Hiromu Murakoshi, Kyoko Hiragami-Hamada, Reiko Nakagawa, Wolfgang Fischle, Yoshifumi Nishimura, Hitoshi Kurumizaka, Hideaki Tagami, and Jun-ichi Nakayama

**SUPPLEMENTARY METHODS**

**CK2α/α’ knock-down**

For CK2 knock-down experiments, NIH3T3 cells were transfected with 100 pmol of control siRNA cocktail (B-Bridge International), CK2α ON-TARGETplus SMARTpool siRNA (L058653, Dharmacon), and/or CK2α’ ON-TARGETplus SMARTpool siRNA (L051582, Dharmacon) in a 12-well plate using Lipofectamine RNAiMAX (Invitrogen). After 72 h, the siRNA-treated cells were harvested and lysed in 1x SDS sample buffer. The level of CK2αα’ in each cell population was determined by immunoblotting using an anti-CK2α/α’ antibody (Calbiochem). An anti-Lamin B antibody (sc6217, Santa-Cruz) was used as a loading control. The phosphorylation of HP1α in the siRNA-treated cells was analyzed by Phos-tag-PAGE and Western blotting using an anti-HP1α antibody.

**Treatment of cultured cells with CK2 inhibitors**

Various concentrations of 4,5,6,7-tetrabromobenzotriazole (TBB; Calbiochem) were added to NIH3T3 cell cultures that had been grown to approximately 70% confluency, and incubated for 3, 16, or 24 h. After the incubation, the cells were lysed in RIPA buffer supplemented with protease inhibitor cocktail and 50 mM NaF, and the phosphorylation of endogenous HP1α was analyzed by Phos-tag-PAGE and Western blotting using an anti- HP1α antibody. To further examine the effect of a CK2 inhibitor on newly synthesized HP1α, T-REX HeLa cells expressing FLAG-tagged HP1α (FLAG-HP1α) under the control of tetracycline/doxycycline were isolated and maintained in MEM (Nakalai Tesque) supplemented with 1 mM sodium pyruvate, 10% fetal calf serum (Gibco), and 2 µg/mL puromycin (Invivogen). The endogenous CK2 activity was inhibited with 50 µM tetrabromocinnamic acid (TBCA; Calbiochem), and the FLAG-HP1α expression was induced by adding 0.5 µg/ml doxycycline at various time points before harvesting (-9, -12, -18, or -24 h). The phosphorylation of newly synthesized FLAG-HP1α was analyzed by Phos-tag-PAGE and Western blotting using an anti-FLAG M2 antibody.

**
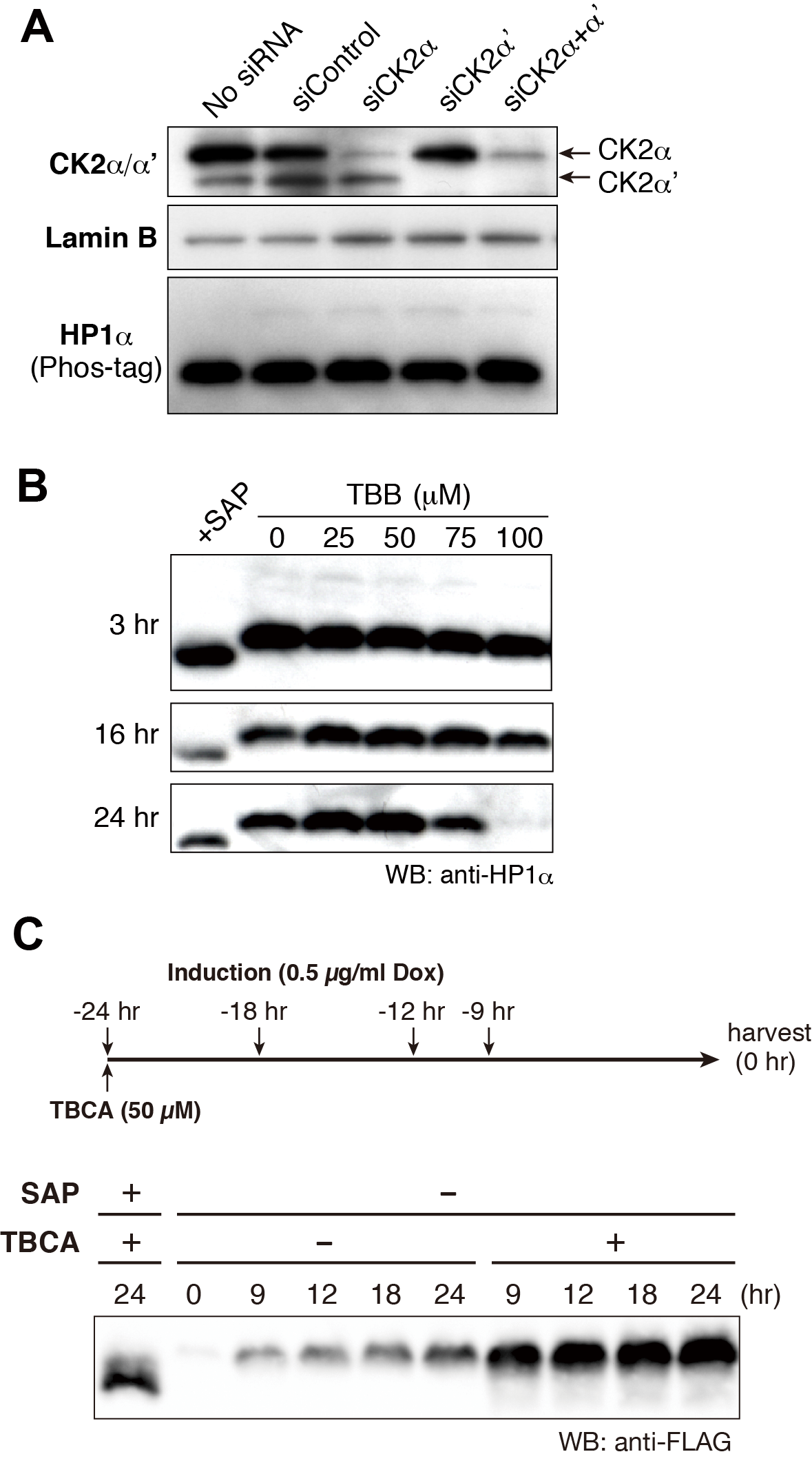
**

**Supplementary Figure S1. Effects of CK2 knockdown or inhibitors on HP1α phosphorylation**.

(A) Effect of CK2α/α’ knockdown on the phosphorylation of HP1α *in vivo*. NIH3T3 cells were treated with control siRNA or siRNA against CK2α and/or CK2α’catalytic subunits for 72 h. Whole cell lysates (WCLs) were prepared from each cell population, and the phosphorylation of endogenous HP1α was analyzed by Phos-tag PAGE and Western blotting using an anti-HP1α antibody. An anti-Lamin B antibody was used as a loading control. (B) Effect of TBB on the *in vivo* phosphorylation of HP1α. ΝΙΗ3Τ3 cells were grown in the presence of the indicated concentrations of TBB for 3, 16, and 24 h. The phosphorylation of the endogenous HP1α was analyzed as in (A). An aliquot of the WCL from TBB-untreated cells was treated with alkaline phosphatase (+SAP) as an unphosphorylated control. Note that the cell growth was arrested at higher concentrations (75, 100 μM) of TBB. (C) Effect of TBCA on the *in vivo* phosphorylation of newly synthesized HP1α. HeLa cells expressing inducible FLAG-HP1α were treated with TBCA, and the HP1α expression was induced at various time points. The phosphorylation of the induced FLAG-HP1α was analyzed by Phos-tag PAGE and Western blotting using an anti-FLAG antibody.

**
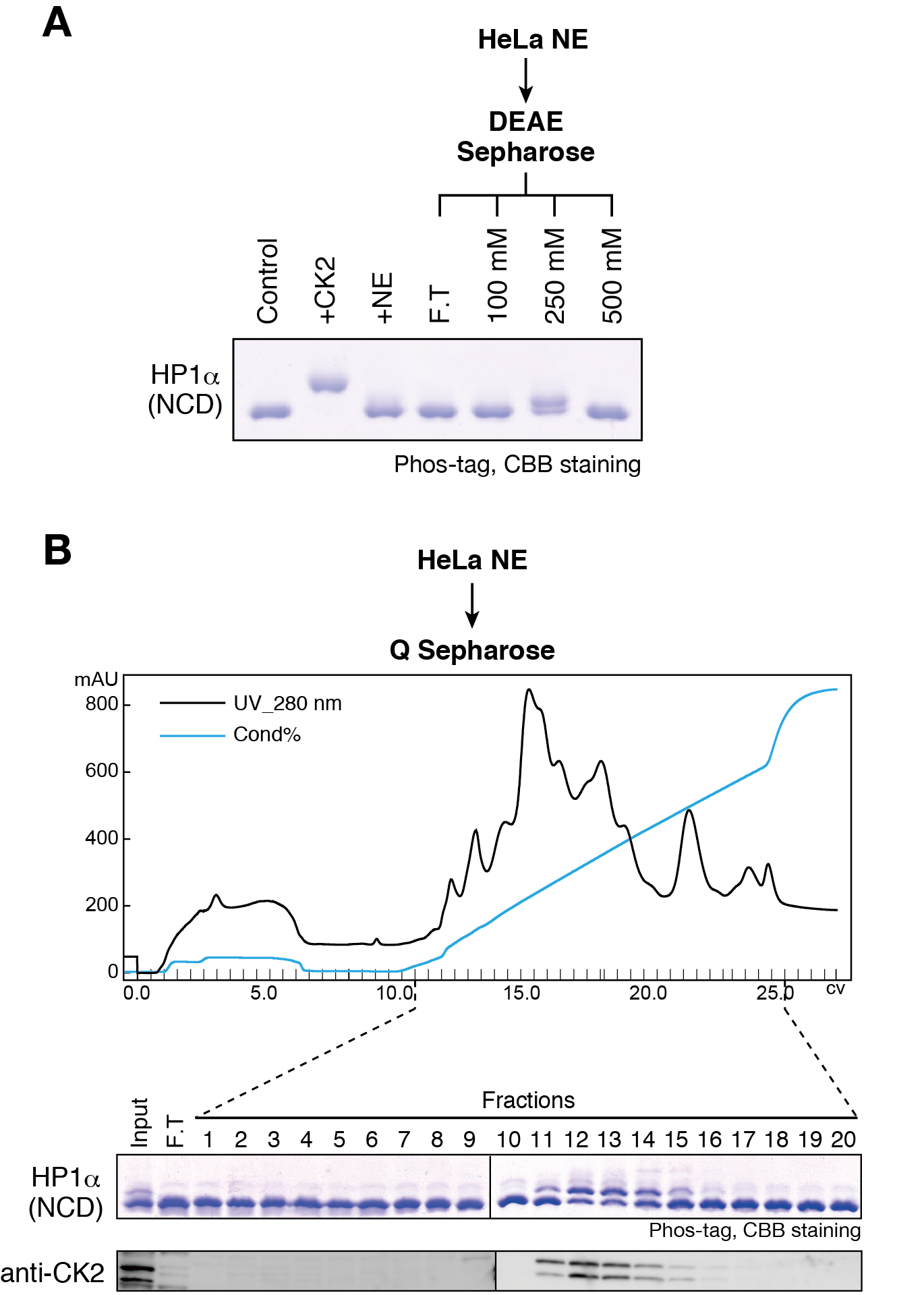
**

**Supplementary Figure S2. HP1α is constitutively phosphorylated by CK2 at N-terminal serine residues in human cells**.

(A) An *in vitro* kinase assay using HP1α-NCD. HeLa nuclear extracts (NE) and fractions partially purified through DEAE Sepharose were assayed. Resultant samples were resolved on Phos-tag PAGE and visualized by CBB staining. (B) The partial purification of cellular kinase(s) that phosphorylate HP1α-NCD. HeLa NEs were loaded onto a HiTrap Q HP Column, and each fraction was subjected to a kinase assay. Elution profiles of endogenous CK2 were analyzed by Western blotting using an anti-CK2α antibody (bottom).

**
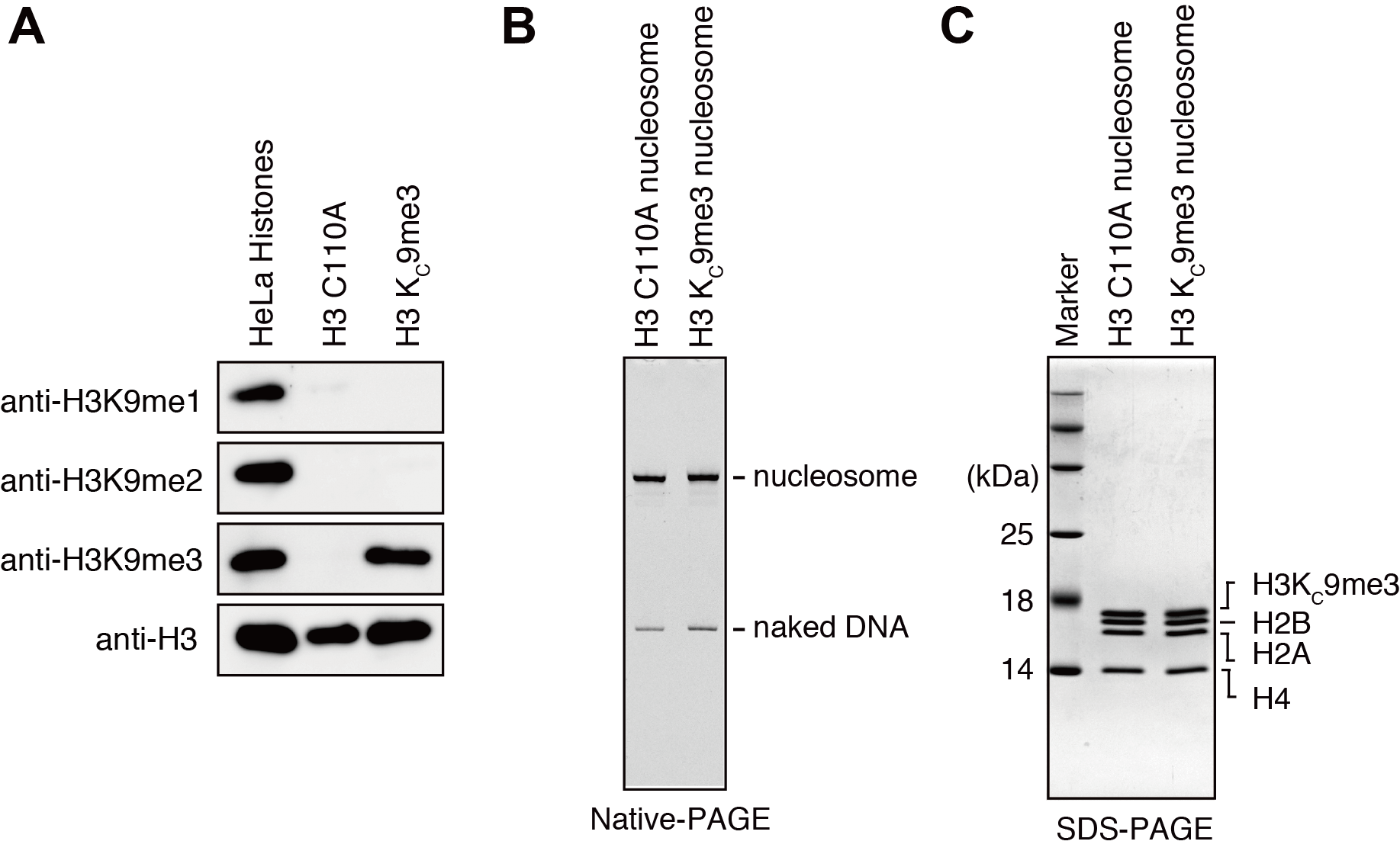
**

**Supplementary Figure S3. Characterization of reconstituted nucleosomes.**

(A) Western blotting analysis of the H3K9me3 analog. Purified recombinant histone H3.2 C110A and methyl-lysine analog-introduced H3.2 Kc9me3 (C110A) were analyzed by Western blotting using anti-H3K9me1, -H3K9me2, -H3K9me3, or -H3 (whole) antibodies. Histones prepared from HeLa S3 cells were used as controls. (B, C) Characterization of reconstituted nucleosomes. Reconstituted nucleosomes were analyzed by native-PAGE (B) and SDS-PAGE (C) and visualized by ethidium bromide and CBB staining, respectively.

**
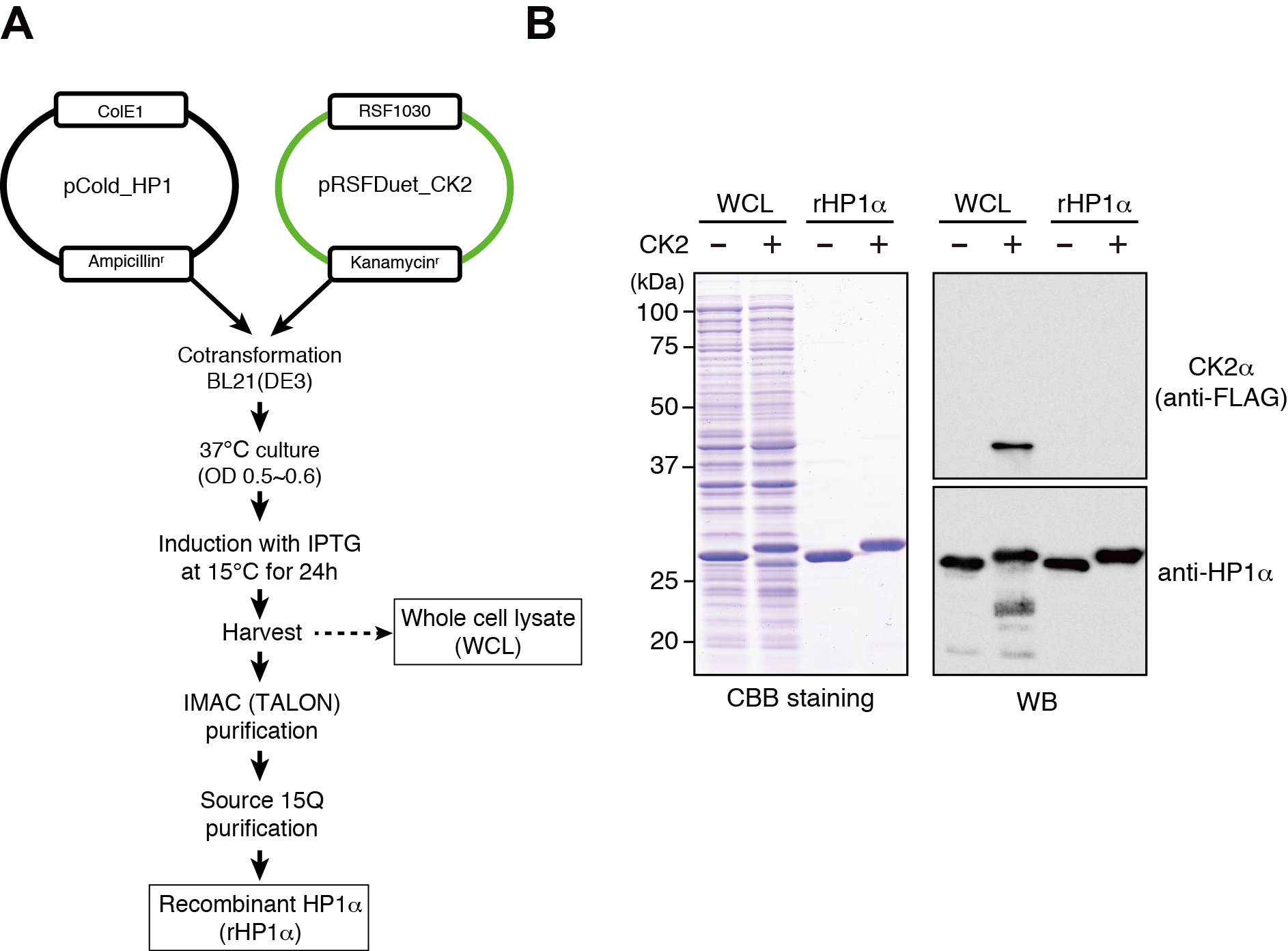
**

**Supplementary Figure S4. Preparation of CK2-phosphorylated recombinant HP1α.**

(A) Experimental scheme for preparing CK2-phosphorylated recombinant HP1α. BL21 (DE3) *E. coli* cells were simultaneously transformed with two plasmids expressing HP1α (pCold-HP1) and the α and β subunits of CK2 (pRSFDuet-CK2), and selected with medium containing both ampicillin (for pCold l) and kanamycin (for pRSFDuet). (B) Characterization of purified HP1α. *E. coli* whole cell lysates (WCLs) and purified recombinant HP1α proteins (rHP1αs) were subjected to SDS-PAGE and analyzed by CBB staining (left panel) or by Western blotting using an anti-FLAG antibody for CK2α (right, top panel) or an anti-HP1α antibody (right, bottom panel).

**
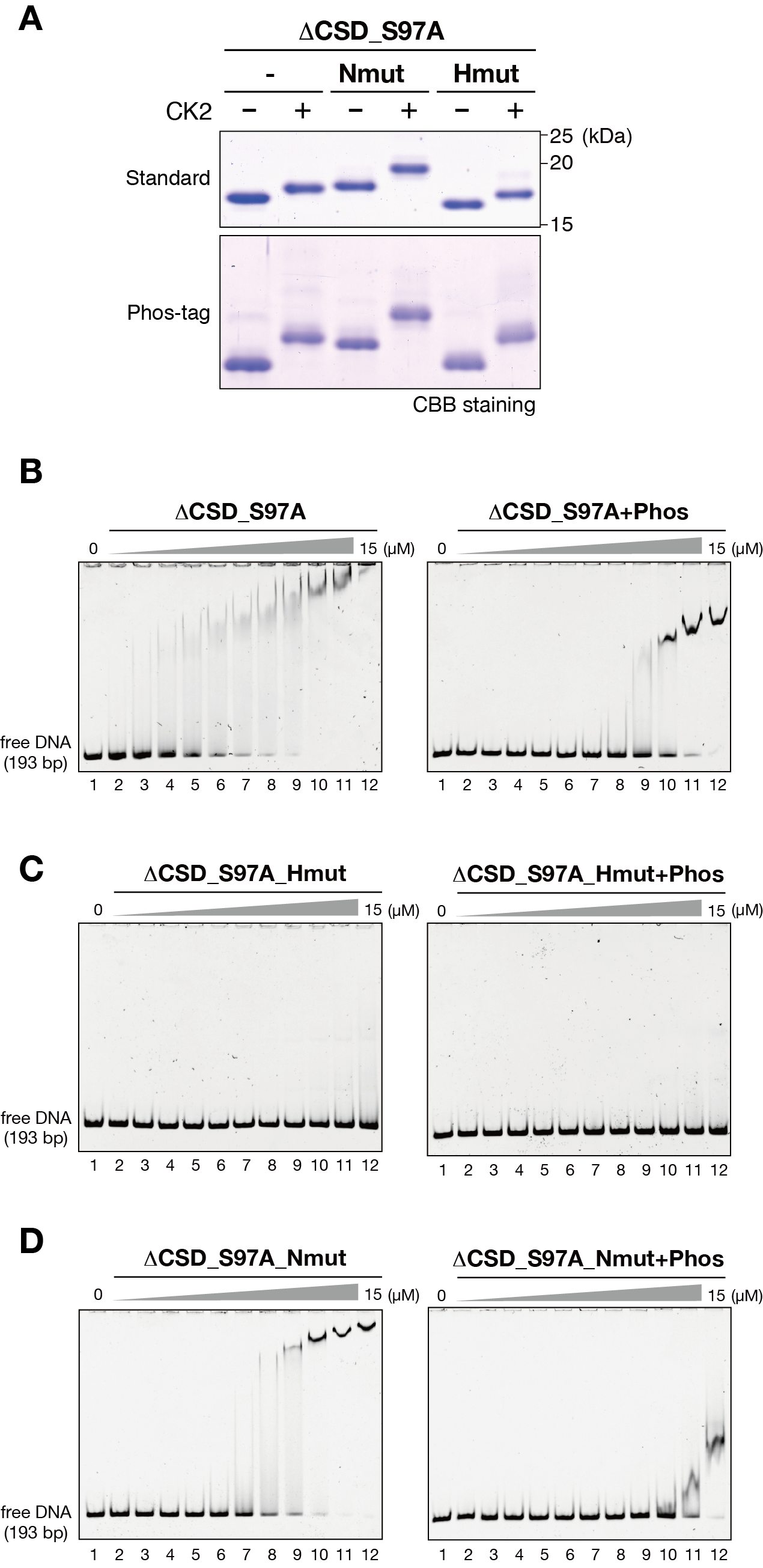
**

**Supplementary Figure S5. Basic amino acid residues at the N-terminal tail modulate hinge region-mediated DNA binding.**

(A) Control and CK2-phosphorylated ∆CSD_S97A, ∆CSD_S97A-Nmut, and ∆CSD_S97A-Hmut proteins were resolved by standard or Phos-tag PAGE and visualized by CBB staining. (B-D) Representative EMSAs using control and CK2-phosphorylated ∆_S97ACSD (B), ∆CSD_S97A-Hmut (C), and ∆CSD_S97A-Nmut (D). The protein concentration varied from 0 to 15 µM (0.5-fold dilutions). A 193-bp 601 DNA was used as a probe.
